# The Potential Role of Vitamin D in the Development of Tuberculosis in Chinese Han Population: One Case-Control Study

**DOI:** 10.3389/fmed.2022.849651

**Published:** 2022-07-25

**Authors:** Li Cai, Shuangyi Hou, Yadong Huang, Shuang Liu, Xibao Huang, Xiaoxv Yin, Nan Jiang, Yeqing Tong

**Affiliations:** ^1^Wuhan Center for Disease Control and Prevention, Wuhan, China; ^2^School of Public Health, Wuhan University, Wuhan, China; ^3^Center for Disease Control and Prevention, Wuhan, China; ^4^School of Public Health, Tongji Medical College, Huazhong University of Science and Technology, Wuhan, China

**Keywords:** pulmonary tuberculosis, vitamin D, matched case-control study, Han population, role

## Abstract

**Background and aims:**

Spinal serum 25-hydroxyvitamin D [25[OH]D] status plays an important role in mediating innate immune responses by acting as a cofactor for induction of antimycobacterial activity and is thus involved in the development of Tuberculosis (TB). Results reported regarding the association of vitamin D with TB remained controversial. We aimed to identify any common association between 25[OH]D status and TB in the Chinese Han population.

**Methods:**

280 subjects (70 TB patients and 210 matched controls) were recruited. TB cases were diagnosed based on the presence of acid-fast bacilli on smears from sputum and MTB isolation. Healthy controls were randomly selected from four local community-based populations. 25[OH]D was detected by electrochemiluminescence immunoassay (ECLIA) on Roche Elecsys before the initial treatment. Multivariable logistic regression analysis was used to examine the association of Vitamin D with TB.

**Results:**

There was no significant difference in the serum vitamin D level between total cases and controls, but we found a strong tendency toward a higher serum vitamin D level in male population (*P* < 0.05) with TB but not in females. High serum vitamin D increased the risk of TB in the Chinese Han population (OR = 1.035, 95%CI: 1.001–1.070, *P* < 0.05). The serum vitamin D level was significantly decreased with age increasing in cases and controls (all *P* < 0.001).

**Conclusions:**

High serum vitamin D may be an independent risk factor for TB in the Chinese Han population.

## Introduction

Tuberculosis (TB), an essential major social and public health concern worldwide, has received more and more extensive attention. According to the latest WHO reports, TB infection rates were as high as 1/3 in the world population, and 1/10 infected with TB will develop TB disease in the next 10 years, of which, estimated 1/3 are concentrated in Asian countries (1–3). Moreover, China, a Southeast Asian country with high TB prevalence and incidence in the population, has already begun to focus on TB control and prevention. Concentrated efforts in China to control the spread of TB resulted in a dramatic decline in TB incidence between 1990 and 2010 (4–6). However, recent statistics still indicated about 1 million new TB cases per year in China (7–9).

In these years, an increasing number of studies have indicated vitamin D was associated with the risk of TB (10–12) and other infectious diseases such as respiratory tract infections (RTI) (13), human immunodeficiency virus (HIV) (14), fungal infections (15), and sepsis (16). Vitamin D plays a significant role in the innate defenses against intracellular pathogens. The active metabolite, 1,25-dihydroxyvitamin D3 [1,25(OH)_2_D_3_], has been shown to improve the ability of macrophages to inhibit the growth of Mycobacterium tuberculosis (MTB) through stimulating the cell-mediated immunity monocyte/macrophage pathway (17, 18). Wilkinson found that a lower level of 25(OH)D may increase the susceptibility of TB (19). However, a population study in Indonesia showed that the 25(OH)D level was not associated with the risk of active TB (20). Nevertheless, a meta-analysis concluded that low serum vitamin D concentrations were associated with an increased risk of active TB (21). Thus, to date, the evidence on the relationship between vitamin D and TB remains controversial. Although studies on the association of vitamin D with TB have been conducted in some European countries, few studies have been conducted in Asian countries, especially in China.

Therefore, given the correlated mechanisms of vitamin D in the development of TB and the fact that the association between vitamin D and TB is still poorly understood in China, this study aims to determine how strongly vitamin D contributes to active TB in the Chinese Han population and further assess the association between vitamin D levels and TB progression as well as feasibility of population-based vitamin D supplementation.

## Methods

### Study Design and Subjects

Our study was approved by the Institutional Review Board of Hubei Center for Disease Controls and Prevention, and signed informed consents were obtained from all participants or patients' representatives if direct consent could not be obtained. The experiment methods were carried out in accordance with the approved guidelines and regulations, and all experimental protocols were approved by the Institutional Review Board of Hubei Center for Disease Controls and Prevention.

We conducted a matched case-control study. Each TB case was matched with three non-TB controls to ensure sufficient power to assess any links between vitamin D and TB. We recruited patients who attended the TB Unit of the largest TB hospital in Wuhan from 2014 to 2015. These cases were invited to participate in the study before the anti-TB treatment. A total of 70 typical Han subjects presenting with first-ever TB were included in this case-control study. Concurrently, 210 normal healthy controls matched to the cases in age, gender and ethnicity were randomly selected from four local community-based populations with established medical check-up reports consecutively. Individuals with a history of TB, malabsorption syndrome, human immunodeficiency virus (HIV), and vitamin D deficiency were excluded from the control group.

The diagnosis of TB was established by culturing MTB or acid-fast bacilli (AFB) on smears from sputum. According to the WHO standard, TB cases were diagnosed by the following protocols: (1) two or more different sputum smears are positive, (2) one positive AFB smear and one positive culture; and (3) one positive AFB smear and a typical result of pulmonary TB infection using by chest X-ray. AFB was assessed using Ziehl-Neelsen staining fluorescence microscopy (auramine-rhodamine staining) or fluorescence microscopy (auramine-rhodamine staining) Ziehl-Neelsen staining method. Sputum must contain 5,000 to 10,000 bacilli/mL was considered to be positive. Sputum smear grading was divided into five grades according to the number of AFB: negative (0 AFB/100 high power fields [HPF]), scanty (1-9 AFB/100 HPF), +1 (10-99 AFB/100 HPF), +2 (1-10 AFB/HPF), +3 (>10 AFB/HPF) (22, 23).

### Sample Size Calculation

The sample size was calculated according to the sample size formula for case-control studies (24):


$$\begin{array}{l} n=\;[Z_{\alpha }\sqrt{\left(1+1/r\right)\overset{-}{p}\left(1-\overset{-}{p}\right)}\;\\ {+Z_{\beta }\sqrt{p_{1}\left(1-p_{1}\right)/r+p_{0}\left(1-p_{0}\right)}]}^{2}/{\left(p_{1}-p_{0}\right)}^{2} \end{array}$$

where n is the sample size, Z is the normal deviation, α = 0.05, Z_α_ = 1.96, β = 0.10, Z_β_ = 1.28, *r* = 3. Previous research reported that the probability of exposure in the control group was 11.0%, and the OR of association between exposure to TB was 6.5 (25). After calculation, the minimum sample size required for this study was 22 for the case group and 66 for the control group. Finally, we included 70 and 210 individuals in the case and control groups.

### Vitamin D Estimation

The total serum 25(OH)D concentration for all study participants were detected by electrochemiluminescence immunoassay (ECLIA) on Roche Elecsys 10100/201 system (Roche Diagnosis Elecsys) before the anti-TB treatment. The concentration of 25(OH)D ranging from 10 to 250 nmol/L could be measured by ECLIA sensitively. The assay sensitivity is 3.75 nmol/L with an intra-assay CV of 5.6% at 39.75 nmol/L and 11.6% at 147.25 nmol/L. The inter-assay CV at these two levels was 9 and 12%, respectively. We defined vitamin D insufficiency, mild deficiency of vitamin D, and severe deficiency of vitamin D as serum 25(OH)D concentrations of 50–75 nmol/L, 25–49 nmol/L and <25 nmol/L, respectively, according to the previously used definitions (26, 27). Serum 25(OH)D concentrations of 76–140 nmol/L and above 140 nmol/L were featured as normal and high, respectively (25). Serum 25(OH)D concentration ≤75 and >75 nmol/L were deemed as vitamin D insufficient and sufficient for individuals, respectively.

### Statistical Analysis

Data on continuous variables were presented by means and standard deviations because they were normally distributed. We checked the normality of the distribution for quantitative variables by the Kolmogorov–Smirnov test and the Shapiro–Wilk test. Data on category variables were presented by number and percentage. Comparisons of continuous variables and category variables among groups were made by Student's *t*-test or one-way analysis of variance (ANOVAs) and Chi-square test, respectively. Multivariable analysis was performed using binary logistic regression model to analyze the association between vitamin D status and the risk of TB. The odds ratio (OR) and 95% confidence intervals (CIs) for independent variables were reported. All statistical analyses were performed using the SPSS 12.0 software package (SPSS Inc., Chicago, Illinois, USA). For all analyses, all *P*-values were two tailed, and differences were considered point-wise statistically significant for *P* < 0.05.

## Results

A total of 70 TB patients and 210 healthy controls were evaluated for the association of serum vitamin D level with TB from Han subject panel of China. The ratio of males to females was 93:117 in the Han subjects. The average age of cases and controls were 39.99 ± 7.87 and 39.97 ± 18.00, respectively. There were no significant differences in age between cases and controls in the Han population (*P* > 0.05). All the cases and controls were not HIV-infected. The sputum smear grading results are shown in Supplementary Table S1.

**Figure 1:**
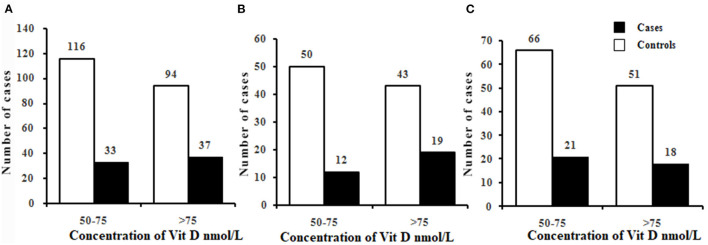
Prevalence of vitamin D insufficiency in cases and controls in the total **(A)**, male **(B)**, and female **(C)**. Chi-square tests were used to compare the differences in the prevalence of vitamin D insufficiency between cases and controls. There were no significant differences in the prevalence of vitamin D insufficiency between cases and controls in the total, male, and female (all *P* > 0.05). Vitamin D groups: vitamin D insufficiency (50–75nmol/L); vitamin D sufficiency (>75nmol/L).

The serum levels of 25(OH)D were 75.71 ± 10.75 nmol/L and 73.39 ± 10.01 nmol/L in total cases and controls, respectively, and there was no significant difference in 25(OH)D levels between the two groups (*P* > 0.05) (Table 1). Using the criteria of 25(OH)D ≤75 nmol/L, the prevalence of vitamin D insufficiency were 47.14 vs. 55.24%, 38.71 vs. 53.76% and 53.84 vs. 56.41% in total, male and female population. It seems that this prevalence has a lower trend in cases than in controls. The further precise stratified analysis by vitamin D level insufficiency showed that there were no significant differences between the cases and controls among the male, female and total population (all *P* > 0.05) (Figure 1). However, by quantitative analysis, serum levels of 25(OH)D were higher in male patients with TB than in male control population (*P* < 0.05). In female population, there was no significant difference in 25(OH)D between TB patients and controls (*P* > 0.05).

**Table 1:** The distributions of vitamin D level in different sex population.

	**Cases**		**Controls**		***t*-value**	***P*-value**
	***N***	**Vit D (nmol/L)**	***N***	**Vit D (nmol/L)**		
Sex
Male	31	78.81 ± 10.81	93	72.67 ± 10.88	−2.49	**<0.05**
Female	39	73.26 ±10.17	117	73.96 ± 9.27	0.40	0.690
*t*-value	1.99	−0.91	–	–
*P*-value	0.051	0.363	–	–
Total	70	75.71 ± 10.75	210	73.39 ± 10.01	−1.49	0.137

*The bold value indicates a statistical difference between case and control groups at the significance level of 0.05*.

To further find the potential association of serum vitamin D level with TB in the Chinese Han population, we also analyzed the association of serum vitamin D level with TB in different age groups. However, we failed to find a significant association between the above two in any age group (all *P* > 0.05) (Table 2). It is worth noting that serum vitamin D level was significantly decreased with age increasing in cases and controls, respectively (all *P* < 0.001).

**Table 2:** The distributions of vitamin D level in case and control population stratified by age.

	**Cases**		**Controls**		***t*-value**	***P*-value**
	***N***	**Vit D (nmol/L)**	***N***	**Vit D (nmol/L)**		
Age (Y)
15~24	19	81.16 ± 8.88	68	79.87 ± 7.17	−0.66	0.513
25~44	22	78.45 ± 8.66	53	76.57 ± 5.39	−0.95	0.350
45~59	17	72.53 ± 10.87	51	69.55 ± 10.11	−1.03	0.305
>60	12	66.58 ± 10.36	38	62.50 ± 7.76	−1.46	0.150
*F*-value	6.90	47.22	–	–
*P*-value	<0.001	<0.001	–	–
Total	70	75.71 ± 10.75	210	73.39 ± 10.01	−1.49	0.137

A multivariable logistic regression model analysis was performed, which may provide more insight into the relationship between vitamin D levels and TB than the univariable analysis could reveal. After adjustment for confounders, there was still a significant difference in vitamin D levels among cases and controls (*P* < 0.05). Compared with low vitamin D levels, the risk of TB was increased 1.04 times with high vitamin D levels in the Han population. It is suggested that high vitamin D levels may be an independent risk factor for TB in the Han population (Table 3).

**Table 3:** Risk of TB and Vitamin D in the Han population by logistic regression analysis.

**Variables**	**Coefficient**	**SE**	**Wald χ^**2**^**	**OR (95%CI)**	***P*-value**
Sex	−0.022	0.280	0.006	0.978 (0.565–1.695)	0.938
Age	0.011	0.009	1.429	1.011 (0.993–1.030)	0.232
Vit D	0.034	0.017	4.157	1.035 (1.001–1.070)	**<0.05**

*Multivariable logistic regression analysis after adjustment for age and sex*.

*CI, confidence interval; TB, tuberculosis; OR, odds ratio*.

*The bold value indicates high vit D levels had a significant effect on TB*.

## Discussion

There is a growing recognition that vitamin D was associated with immunity, resulting from many previous *in-vitro* studies. But the studies *in-vivo* on the association of vitamin D status with TB have yielded inconsistent findings. Davies et al. *first* reported that serum concentrations of vitamin D were associated with untreated TB 20 years ago (28). Most studies indicated that TB had a lower 25(OH)D level and a higher prevalence of vitamin D deficiency than those non-TB controls. However, subsequent studies showed conflicting or different results. A number of studies in sub-Saharan Africa, Birmingham, US, Mwanza, Tanzania, Tanzania, Malawi, and London populations have drawn different significant associated conclusions (29–37). However, the Indonesia and Hong Kong studies (20, 35) failed to find a significant association between vitamin D levels and TB. More interestingly, both high and low serum vitamin D concentrations were explicitly associated with TB in the Greenland population (25). We speculated that differences in confounding factors at adjustment (38), genetic polymorphisms of vitamin D receptors in the study population (19), study designs (39, 40), and vitamin D measurement methods (41) in studies from different countries contributed to inconsistent findings. In the current study, it is worth noting that vitamin D levels were significantly higher in males but not in females, which was broadly inconsistent with most previous studies.

In addition, whether vitamin D is associated with TB by gender is an interesting focus. From our study, we found that serum 25(OH)D levels were higher in male patients than in male controls, but not in the female population, which was consistent with the conclusion of a West African study that vitamin D was associated with TB in the male population only (42). The West African study found a lower prevalence of vitamin D insufficiency in female cases than in female controls (42). However, the reasons why the association have sex-specific characteristic remain unclear yet. Some studies have reported that the difference in the amount of subcutaneous fat between males and females makes females more susceptible to vitamin D insufficiency than males (43, 44). Therefore, an association between high levels of vitamin D and TB could easily be observed in males in this study. Nevertheless, more populations are warranted to validate this interesting observation finding.

The potential mechanisms of this observed association may be explained as that high 25(OH)D concentration may activate the 24-hydroxylase (CYP24A1), degrading 25(OH)D to 24,25(OH)_2_D, which is an inactive form of vitamin D (14). This conversion process would result in low 25(OH)D concentrations. Evidence has suggested that a fall in serum 25(OH)D levels compromises cell-mediated immunity and leads to the activation of latent TB (21). Alternatively, increased 25(OH)D concentration might cause a down-regulation of vitamin D receptor expression resulting in defective vitamin D receptor signal as previous studies have shown (45–47). However, the active form of 25(OH)D is mediated through the vitamin D receptor (VDR) to enhance macrophage activity and increase the production of cathelicidin, particularly the promotion of antimicrobial peptide LL-37 production, one of the defensins-antimicrobial peptides of the cathelicidin family involved in the TB destruction (17, 18, 48–54). Through these potential mechanisms, high vitamin D levels may lead to increased susceptibility to TB.

Our study has certain strengths, including a relatively large sample size in the Han population, rigorous methods used to diagnose TB, including culturing MTB or acid-fast bacilli (AFB) and X-ray. Our population was highly homogenous concerning ethnicity and geographic regions. In addition, vitamin D estimation was precisely detected by the ECLIA technique, which has high sensitivity and specificity as HPLC and other traditional methods (55). However, this study also has some limitations, similar to any other epidemiological study. First and foremost, this is a matched case-control study, and selection bias cannot be excluded from the TB patient group. Also, the temporal sequence between exposure and disease outcome is difficult to determine. Further prospective design studies are needed to verify the causal correlation between high vitamin D levels and TB. Second, it is possible that our findings only apply to the Chinese Han population. Third, we did not measure serum calcium and parathyroid hormone (PTH) in this study because no direct association was observed between these two and vitamin D in Chinese studies (56, 57).

In conclusion, increased 25(OH)D levels may be an independent risk factor for TB in the Chinese Han population, suggesting that vitamin D may be a predisposing marker for assessing TB susceptibility risk. Population-based vitamin D supplementation might not be suitable in the Chinese Han population, as supplementation of non-deficient individuals may result in a high vitamin D concentration, leading to a higher risk of TB. Further explorations with larger, more ethnically diverse populations are warranted to better shed light on the functional properties of vitamin D on the risk of TB, as well as the complicated pathophysiological mechanisms precisely. These valuable insights may affect the diagnosis and treatment of TB.

## Data Availability Statement

The original contributions presented in the study are included in the article/Supplementary Material, further inquiries can be directed to the corresponding author/s.

## Ethics Statement

The studies involving human participants were reviewed and approved by Wuhan Local Ethics Committee. The patients/participants provided their written informed consent to participate in this study.

## Author Contributions

YT, LC, and SH conceived and designed the experiments. YT, LC, XH, and XY performed the experiments. YT, SL, and YH analyzed the data. YT, LC, XH, and YH contributed reagents, materials, and analysis tools. YT, XH, XY, and NJ wrote the paper. All authors contributed to the article and approved the submitted version.

## Funding

This study was supported by funds (92169117) from National Science Foundation of China and the young Top-notch Talent Cultivation Program of Hubei (2021) and Hubei young Top-notch Talent project (2019&2021) as well as Hubei outstanding young Science Foundation of Hubei (2020CFA075).

## Conflict of Interest

The authors declare that the research was conducted in the absence of any commercial or financial relationships that could be construed as a potential conflict of interest.

## Publisher's Note

All claims expressed in this article are solely those of the authors and do not necessarily represent those of their affiliated organizations, or those of the publisher, the editors and the reviewers. Any product that may be evaluated in this article, or claim that may be made by its manufacturer, is not guaranteed or endorsed by the publisher.
