# Supplementary material for: The Potential Role of Vitamin D in the Development of Tuberculosis in Chinese Han Population: One Case-Control Study
Source: Front Med (Lausanne). 2022 Jul 25;9:849651. doi: 10.3389/fmed.2022.849651 (PMC9358990; doi:10.3389/fmed.2022.849651)
Supplement: Supplementary file 1 [file Table_1.DOCX]

**Table S1.** The grading of smear in TB patients.

| Grading of smear | Number | % |
| --- | --- | --- |
| Negative | 0 | 0 |
| Scanty | 0 | 0 |
| +1 | 0 | 0 |
| +2 | 0 | 0 |
| +3 | 70 | 100 |
